# Techno-Functional and Gelling Properties of Acha (Fonio) (*Digitaria exilis stapf*) Flour: A Study of Its Potential as a New Gluten-Free Starch Source in Industrial Applications

**DOI:** 10.3390/foods11020183

**Published:** 2022-01-11

**Authors:** Aloisa G. Deriu, Antonio J. Vela, Felicidad Ronda

**Affiliations:** 1 Department of Agriculture and Forestry Engineering, Food Technology, PROCEREALTech Research Group, University of Valladolid, 34004 Valladolid, Spain; deriualoisa@gmail.com (A.G.D.); antoniojose.vela@uva.es (A.J.V.); 2 Department of Agriculture, Section of Environmental and Food Sciences and Technologies, University of Sassari, 07100 Sassari, Italy

**Keywords:** fonio, gelation, gel texture, gel viscosity, rheological properties, thermal properties

## Abstract

Fonio (*Digitaria exilis Stapf*) is an ancient African cereal that represents a rich source of carbohydrate, fat, fiber, vitamins, minerals, and sulfur-containing amino acids. Processing and utilization of fonio require adequate knowledge of its structural, chemical, and nutritional characteristics. The present work evaluates the structural, techno-functional, and gelling properties of fonio and compares them to other major gluten-free cereals (rice, maize, sorghum, and millet). Fonio flour presented significantly higher water absorption index and swelling power, while it scored a lower water solubility index than the reference flours. The pasting viscosity profile of fonio was similar to that of rice, with equivalent peak viscosity but a breakdown viscosity 24% lower than rice, indicative of higher stability and resistance to shearing and heating. Rheological properties demonstrated that fonio generates gels with remarkably strong structures. At 15% concentration, fonio gel withstood stress 579% higher than those observed in the reference flours without breaking its structure. Fonio flour presented the highest gelatinization enthalpy (11.45 J/g) and a narrow gelatinization temperature range (9.96 °C), indicative of a better-packed starch structure than the other analyzed flours. The texture of the gels made with fonio showed higher firmness over the evaluated period. These combined results suggest that fonio is a suitable ingredient for gel-like food formulations.

## 1. Introduction

Over the last decade, the market for gluten-free (GF) products has grown considerably as a consequence of better diagnostic methods for identifying an increasing number of people suffering from celiac disease and other gluten-related disorders, and people who have eliminated gluten from their diet because they perceive it as a healthy improvement [1]. The nutritional composition of gluten-free products can be the most important cause of macro and micronutrient deficiencies in people with celiac disease [2]. For this reason, there are many concerns about the nutritional inadequacy of the gluten-free diet, often characterized by an excess of calories and a reduced intake of fiber, minerals, and complex carbohydrates [3,4]. It is very important for gluten-free diets to be balanced and diverse, making it necessary for more gluten-free sources to be studied and applied in the development of novel food products with improved sensorial quality and nutritional value.

*Digitaria exilis Stapf*, also known as fonio or acha, is a naturally gluten-free African cereal suitable for use in the diet of celiac patients [5]. Despite its low agronomic yield potential, fonio is gaining importance as a crop and food ingredient due to its superior nutritional characteristics compared with other cereals, the increased market interest in traditional food, and its suitability to be grown in tough conditions, such as in arid soil. Fonio flour has been mixed with other ingredients to improve the nutritional and textural quality of different food products such as malts, beverages, sourdough, bread, puddings, crackers, breakfast cereals, and biscuits [6,7].

Fonio has a high content of calcium and iron, compared to the other cereals indicated in the food composition table of Mali [8]. Potassium and magnesium appear to be the major mineral elements in fonio grains [6]. The protein content of fonio is like that found in white rice [9,10], although it has a higher sulfur amino acid content (methionine and cystine [11]. Fonio has a high pentosan content, which gives it the capacity to absorb water to produce a very viscous solution, an attribute known for good baking operation [12]. Fonio has also been linked to health benefits such as the prevention and treatment of constipation, cardiovascular diseases, and hypertension [13]. The presence of polyphenols in fonio leads to antioxidant and free radical scavenging activities [6]. It is believed that fonio may have nutraceutical properties with a role in preventing and managing prediabetes and type 2 diabetes [5]. Sartelet et al. [14] have demonstrated the presence of apigenin and luteolin in fonio manifest strong anti-thyroid peroxidase (TPO) activities. Given these characteristics, fonio flour has the potential to be used as an ingredient to improve gluten-free nutritional profiles without compromising the taste and quality of products.

Despite the nutritional interest and health benefits of fonio flour, there is a lack of research on fonio compared to other major cereal grains [6]. An in-depth study into the techno-functional and gelling properties of fonio has not been covered in the available literature so far. The aim of this work is to evaluate the techno-functional, pasting, and thermal properties of fonio flour and the rheological and textural characteristics of the gels made from it. Other major gluten-free cereals, such as maize and rice (the two main gluten-free ingredients used in Europe), millet and sorghum (two warm-season grains that belong to the same family (*Poaceae*) and sub-family (*Panicoideae*) as fonio), were included in the study as reference flours.

## 2. Materials and Methods

### 2.1. Material

Commercial fonio (*Digitaria exilis stapf*) flour was obtained from Obà Food (Roma, Italy). Maize (*Zea Mais* L.) flour was purchased from ADPAN (Asturias, Spain). Indica rice (*Oryza sativa* L.) flour was kindly provided by Herba Ricemills S.L.U. (Valencia, Spain). Sorghum (*Sorgum bicolor*) and millet (*Panicum miliaceum*) flours were kindly supplied by Salutef (Palencia, Spain).

### 2.2. Flour Proximate Composition

The flours’ composition was determined following the 44–19 (moisture), 08–01 (ash), 30–25 (fat), and 46–11 (protein) AACC methods [15]. Carbohydrates were determined by difference to 100%.

### 2.3. Techno-Functional Properties

Foaming capacity (FC) and foam stability (FS) were determined as described by Abebe et al. [16]. The flour sample (2 g) was mixed with 40 mL of distilled water at 30 °C in a 100 mL measuring test tube. To produce foam, the suspension was shaken manually for 5 min. The volume of foam was measured after 0 min (V_0_) and 60 min (V_60_). FC was established directly from V_0_, and FS was calculated as (V_60_/V_0_) 100.

Water absorption capacity (WAC), water absorption index (WAI), water solubility index (WSI), and swelling power (SP) were determined evaluating dispersions of 2 g of flour sample in 20 mL of distilled water using 50 mL centrifuge tubes, following the methods indicated by Abebe, Collar, and Ronda [16]. The WAC was determined by the centrifugation method. The dispersions were kept at room temperature for 30 min, then vortexed for 30 s and finally left to rest for 10 min. Immediately after, the tubes were centrifuged for 25 min at 3000× *g*. The supernatant was removed, and the sediment was weighed. Results were expressed as g H_2_O retained/g of flour dry matter.

WAI, WSI, and SP were determined after cooking the flour dispersions for 15 min in a 90 °C water bath. The gels formed were cooled at room temperature for 1 h and centrifuged at 3000× *g* for 10 min. The supernatant was placed into an evaporating capsule to determine the soluble solid content (WSI (g of soluble solids/100 g flour dry matter) and SP (g of sediment/g insoluble solids flour dry matter)). The sediment was used to determine WAI (g of sediment/g of dry flour matter).

### 2.4. Least Gelation Concentration

The least gelation concentration (LGC) of the studied flours was determined with slight modifications of the method indicated by Joshi et al. [17]. Glass test tubes containing 2 mL of distilled water and the corresponding amount of flour to achieve a concentration of 2, 4, 6, 9, 10, 11, 12, 14, 16, 18, and 20 g/100 mL were kept for 1 h in a boiling water bath to form the gel. The tubes were cooled down by placing them under running water and stored at 4 °C for 2 h. LGC was determined as the minimum concentration where the gel did not drop or slip when the glass tube was inverted.

### 2.5. Pasting Properties

Pasting properties of flour samples were determined using a Kinexus Pro+ rheometer (Malvern Instruments Ltd., Malvern, UK) equipped with a starch cell and controlled by rSpace software. Each flour sample (3.0 g, dry basis) was transferred to the evaluation canister and mixed with 25 mL of distilled water. The sample was equilibrated at 50 °C for 60 s, heated to 95 °C at 6 °C/min, maintained at 95 °C for 300 s, cooled to 50 °C at 6 °C/min, and maintained at 50 °C for 120 s. The paddle speed rate was set at 160 rpm during the whole analysis. Parameters calculated from the pasting profile were pasting temperature (PT), peak viscosity (PV), trough viscosity (TV), breakdown viscosity (BV), final viscosity (FV), and setback viscosity (SV). Each sample was analyzed at least in duplicate.

### 2.6. Rheological Properties of Flour Gels

The gels of fonio, millet, sorghum, maize, and rice were analyzed by a dynamic oscillatory test, performed using a Kinexus Pro+ rheometer (Malvern Instruments Ltd., Malvern, UK) equipped with a parallel plate geometry (40 mm diameter) of serrated surface at a gap of 1 mm. Gel samples were obtained following the method described for the determination of flour pasting properties (Section 2.5). Dispersions of flour in water at different concentrations (6-8-10-12-15 g/100 g) were used to prepare the gels. Once the gel was prepared, it was placed in the plates and was left to rest for 5 min to allow relaxation. The temperature was stabilized at 25 °C. Strain sweeps were performed from 0.1 to 1000% at a constant frequency of 1 Hz to establish the maximum stress (τ_max_) in the Linear Viscoelastic Region (LVR) and the stress at the cross point (G′ = G″) [18]. The limit of the LVR (τ_max_) was identified as the sharp decrease of G′ modulus, which coincided with the sudden increase of tan(δ). Frequency sweeps were made from 1 to 10 Hz at a constant strain of 1% of the LVR. The values G_1_′, G_1_″, and tan(δ)_1_ were obtained from fitting the frequency sweeps experimental data to the power-law model as described by Villanueva, De Lamo, Harasym, and Ronda (2018) [19]; these variables represent the elastic and viscous moduli and the loss tangent, respectively, at a constant strain of 1%. The exponents obtained from the fitting (a, b, and c) quantify the dependence of the elastic and viscous moduli and the loss tangent to the oscillation frequency. The gels and the rheological tests were carried out in duplicate.

### 2.7. Thermal Properties

Thermal transitions (gelatinization, retrogradation, and amylose-lipid complex dissociation) were determined by differential scanning calorimetry (DSC) (DSC-822e, Mettler Toledo, SAE). Samples (~6 mg) were prepared at a 30:70 (*w*/*w*) flour:water ratio in 40 μL aluminum pans. The scan made for the evaluation went from 0 to 115 °C at a heating rate of 5 °C/min, using an empty pan as reference. Zinc and indium were employed to calibrate the calorimeter. The enthalpy change (ΔH, J/g dry basis) and the gelatinization temperatures [onset (To), peak (Tp), and endset (Te)] were recorded. Endothermic transitions of the retrograded starches were determined with a second scan made after 7 days of sample storage at 4 °C, following the same procedure described for the gelatinization evaluation. Each experimental sample was measured at least in duplicate.

### 2.8. Texture of Fresh Gels and Its Evolution with Time

The texture of gels was measured with a TA-XT2 Texture Analyser (Stable Microsystems, Surrey, UK) provided with the software “Texture expert exceed” version 2.63. The gels were prepared in 50 mL centrifuge tubes by heating 28 g of flour dispersion (at a concentration of 15 g/100 g) in a boiling water bath for 30 min, stirring the dispersion every 2–3 min until the boiling temperature was achieved. The tubes were cooled at room temperature and stored for 2, 6, 10, 24, 48, 96, and 192 h at 4 °C. Gels were left at room temperature for 15 min before the analysis of the texture. Measurements were done on gel cylinders of 2.7 cm diameter and 2 cm height. A “Texture Profile Analysis” (TPA) double compression test was performed using a 75 mm diameter aluminum probe (SMSP/75) to suppress 50% depth, at 1 mm/s speed test, with a 30 s delay between compressions. Firmness (N), springiness, cohesiveness, and gumminess (N) were calculated from the TPA graphs.

### 2.9. Statistical Analysis

One-way ANOVA with parametric tests was used for the statistical analysis using Statgraphics Centurion XVIII software (Bitstream, Cambridge, MN, USA). The Levene test was used to check the homogeneity of variances. The normality of the studentized residuals was evaluated with the Kolmogorov–Smirnov test. Fisher’s Least Significant Difference test at 95% confidence intervals (*p* < 0.05) was used to establish significant differences among means.

## 3. Results and Discussion

### 3.1. Flour Proximate Composition

The composition of fonio and the other studied gluten-free flours are presented in Table 1. It can be noted that the composition of fonio flour was very similar to rice flour. Both flours were the richest in carbohydrates and the poorest in proteins, along with maize. Fonio showed the lowest content of fat and ash among the studied flours. Analyzing the literature available for the fonio flour, large variations of the chemical and nutritional composition were found. Ballagoun [20] reported the following ranges in the main fonio flour components: 5.1–11% proteins, 1.3–5.2% fat, 1–6% ash. These differences can be attributed to genetic factors, geographical situation, environmental influences, agronomic characteristics, and analytical methods used in the determination.

### 3.2. Functional Characteristics

Functional characteristics are summarized in Table 2. The studied flours have shown different values of foaming capacity (FC) and foaming stability (FS). The foaming capacity of flours is mainly related to proteins, which form a continuous, cohesive film around the air bubbles in the foam [21]. Fonio did not exhibit any foaming capacity, while millet and sorghum flours exhibited the highest FC and FS, probably due to their higher protein content. It has been reported that protein–carbohydrate interactions in fonio may contribute to the low solubility of fonio proteins [6], resulting in lower availability to interact with water and generate foam. No FS was measured in fonio, given that no foam was formed. The remaining GF flours showed FS values above 50%, suggesting that the native proteins soluble in water are very surface active in these flours [21].

Flour hydration properties varied significantly among GF flours, which could be explained by the different compositions of the flours, mainly protein, fiber, and starch [21]. In order to obtain good quality from alternative materials (such as novel gluten-free sources), it is necessary to know their hydration properties to balance formulations and adequate technological production processes to counteract changes in the rheological properties caused by the substitution of gluten [22]. It has been documented that hydration is a critical factor in many manufacturing processes of cereal-based products such as pasta, couscous, and bread [22]. The WHC, which quantifies the ability of a matrix to absorb and retain water without the influence of external forces, and the WAC, which depends on the source’s susceptibility to form hydrogen bonds between starch, influenced by the hydrophilic parts in carbohydrates and proteins [23], followed a similar trend. Fonio showed average values for a GF flour in these two parameters, above millet, rice, and sorghum, which presented the lowest water binding capacities, and below maize flour. Fonio flour exhibited the highest WAI and SP and the lowest WSI among the other analyzed flours. This behavior denotes the ability of fonio starch to absorb the highest amount of water during gelatinization and swelling in excess water and retain it in the formed gel [24].

Swelling power (SP) followed an identical evolution as WAI, given that both parameters associate the weight of the formed gel with the number of insoluble compounds in the flour and the whole amount of flour, respectively. When the fonio flour gelatinizes, better interaction with water is exhibited, which could be due to the very small dimension of the starch granules, ~8 µm [25]. Said small size leads to a high specific surface area, with consequent higher interaction and absorption of water [26]. The very low solubility of fonio in comparison with the other GF flours (WSI value about 3 times lower than that of rice and 7 times lower than maize) is compatible with its very low mineral content; it also means a very low lixiviation and solubility of amylose during starch swelling [27].

### 3.3. Pasting Properties

**Figure 1:**
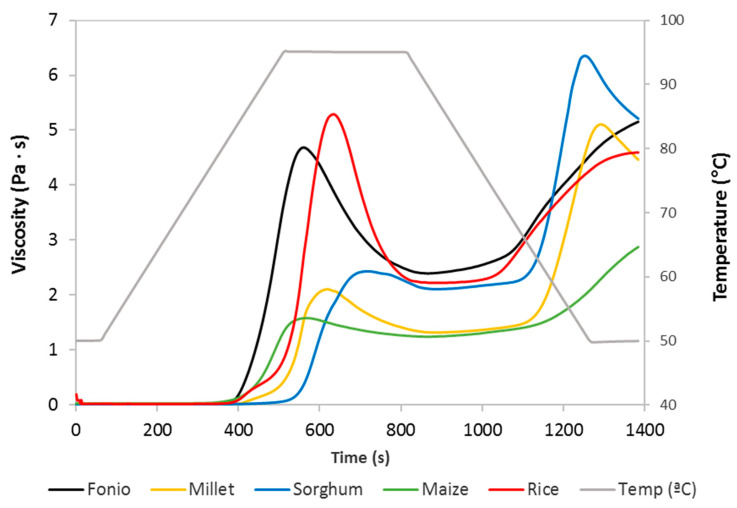
Pasting properties of the gluten-free flours studied.

The results obtained from viscometric tests are presented in Table 2 and Figure 1. Pasting temperature (PT) expressed the minimum temperature necessary to begin the cooking of the flour, identified as the temperature at which viscosity increases during the heating process [28]. Fonio flour showed an intermediate PT value (82.2 °C), similar to that of rice, higher than maize (80.29 °C), and significantly lower than sorghum (91.6 °C) and millet (85.3 °C). The highest value of PT was registered in sorghum flour, which could indicate the presence of a starch that is highly resistant to swelling and rupturing [29]. Fonio showed the shortest time to reach the peak viscosity (Pt) and a peak viscosity (PV) value significantly higher than millet, sorghum, and, especially, maize flour. The PV of fonio flour was only (slightly) surpassed by rice flour. The PV happens at the equilibrium point between swelling and rupturing of the starch [30]. It is obtained at the maximum swelling of starch granules, and it is linked to the water absorption index of the flour [31], as was shown in Section 3.2. Fonio flour also showed a higher trough viscosity (TV) value than millet, sorghum, and maize, similar to that of rice flour. Fonio showed a breakdown viscosity (BV) value 24% lower than rice flour, denoting higher paste stability and resistance against shearing and heating than one of the most used flours in GF production. Maize, followed by sorghum and millet, showed the lowest BV and the highest stability, although these flours presented significantly lower viscometric profiles (Figure 1), so lower values of BV were expected. The setback viscosity (SV) is an indicator of amylose’s tendency to retrograde. In general, higher values of SV indicated a greater tendency of starch to retrograde [32]. The SV value of fonio was similar to that of rice and between those of millet and sorghum (the maximum) and maize (the minimum).

In millet and sorghum, a second peak of viscosity appeared at the final cooling phase (50 °C) of the pasting curve. It is believed that this behavior could be due to the storage of the flours for a time equal to or greater than two months, given that a second peak during the holding stage was also found by Zhang and Hamaker [33] in sorghum flour when it had been stored for more than two months. The viscometric profile of fresh sorghum flour only showed one peak in the holding period (95 °C).

At the end of the pasting profile, during the temperature holding at 50 °C, the final viscosity (FV) was recorded. This value showed the capacity of the material to form a viscous paste that reflects the retrogradation of amylose [34]. The values registered are maximum for sorghum, fonio, and rice flours, and no significant differences are found among them. The lowest FV value was shown by maize flour.

### 3.4. Gel Viscoelastic Properties and Their Dependence on Flour Concentration

The viscoelastic properties of the gels were assessed by dynamic oscillatory tests at 25 °C (see Table 3 and Supplementary Figure S1). The samples were produced at different flour concentrations (from 6% to 15%) and analyzed with strain sweeps, allowing the establishment of the end of the linear viscoelastic region (LVR) and the identification of the maximum stress (τ_max_) that samples could tolerate before the collapse of their structure. The effects of flour type and concentration on the value of maximum stress (τ_max_) were statistically significant (*p* < 0.05), following an increasing trend with increasing concentration in all the studied GF flours. The strain sweep assays also provided the stress at which the gels passed from a solid-like to a liquid-like behavior (the crossing point of the curves where G′ = G″ and tan(δ) = 1) (Table 3). Results indicated that fonio formed gels with a remarkably stronger structure, being the sample that showed the highest values of τ_max_ and cross-over point in all studied concentrations. Differences were particularly marked with higher concentrations; the 15% fonio gel presented a τ_max_ and cross-over point 579% and 1788%, respectively, higher than sorghum (the sample with the lowest results).

The G_1_′, G_1_″, tan(δ)_1_ and a, b and c exponents were generated from fitting the frequency sweeps data in the range of 1–10 Hz to the power-law model. The high value of R^2^ (0.955–0.999) indicates to what extent the model adjusted to the studied systems. Elastic and viscous moduli significantly increased when the flour concentration increased. However, the rate of increase varied depending on the type of flour (see Supplementary Figure S1). Except for the lowest studied concentration (6%), fonio gel exhibited markedly higher elastic and viscous moduli than the other GF flours. The marked differences in gel rheological properties of the different gluten-free flours could be attributed to differences in their botanical origin, such as proteins, starch, and lipids contents [35,36,37].

The viscoelastic moduli of rice gels and the rate of increase of G_1_′ and G_1_″ with concentration were significantly lower than the gels made from the other GF flours, in agreement with previous studies [37].

All gels presented G_1_′ > G_1_″, and consequently tan(δ)_1_ < 1, in all the studied concentrations, indicating a solid-like behavior of the gels. This indicates that the gel structure was already formed at a concentration of 6%, regardless of the flour source. Except for the gels made from rice flour, tan(δ)_1_ decreased with the increase in flour concentration (see Table 3 and Supplementary Figure S1). This behavior reveals a strengthening of their structure with concentration. The fonio gels showed the highest decrease in loss tangent with increasing gel concentration, in particular when increasing from 6% to 8%, reaching the lowest value at a concentration of 12% (0.0540). This value was between 50% and 66% lower than those obtained for the gels made with the other GF flours. In the case of rice gels, the loss tangent did not follow a decreasing trend with concentration, but rather the opposite, indicating that in this case, the increase in concentration enhanced the viscosity of the gel rather than its elasticity, resulting in a softer gel structure than that of the more diluted gels [38].

The low values of “a” exponents for all gels indicate that G′ was not dependent on the applied frequency and suggested a stable gel structure [30]. As can be seen in Table 3, the lowest values of “a” were obtained for fonio gels, while the highest ones were presented by rice gels. The dependence of the viscous modulus and loss tangent with frequency, evaluated from the “b” and “c” exponents, respectively, were higher than that of the elastic modulus (“a”) and similar among gels of different nature. Both exponents decreased with the concentration of flour in the gel, except for rice flour gels, where b increased with concentration. This impact of concentration on the stability of gels and batters has been reported in previous works [30,31,32,33,34,35,36].

The high dependence of storage modulus on the concentration allows gathering information of the gelation efficiency and the structure of the particle network of the gel [39]. Clark et al. [40] estimated the relation between concentration and storage moduli using a power-law equation. Power-law functions between concentration and G_1_′ and G_1_″ were obtained for the dispersions: G_1_′ = m * C^n^ and G_1_″ = *p* * C^q^, where m and p represent the G′ and G″ moduli values at a gel concentration of 1% and a frequency of 1 Hz, and *n* and q, the exponents, quantify the dependence degree of the viscoelastic moduli to the concentration and reflect the nature of the association behavior in the gel and its network structure [41] (Table 4). The R^2^ coefficients ranged from 0.9561 to 0.9997, indicating a good fitting of experimental results to the potential model. In the case of fonio and rice, the evolution of the elastic modulus with concentration would also be compatible with a linear model (see Supplementary Figure S1A). Linear correlations between G′ and concentration have been reported for potato, wheat, corn, and rice starch gels [42]. Therefore, the variation of G_1_′ with the concentration of millet, sorghum, and maize gels, is probably more related to the protein content than to the starch content of the flours, while in the case of fonio and rice, their naturally low protein content could explain the observed behavior, more similar to that of starch gels. However, to allow comparison among different flours, the potential equation was chosen to model the evolution of all GF gels viscoelastic moduli versus concentration. As can be seen (Table 4), the *n* and q values of fonio gels were 2.8 and 2.5, respectively. These values were greater in millet, sorghum, and maize flours, which reflects the formation of a more ordered gel matrix. Rice was the only one with significantly lower exponents (*n* = 1.26 and q = 1.53) than fonio. This denotes a higher increase in fonio gels’ viscoelastic moduli with flour concentration and a higher modulation capacity of the gel’s viscoelasticity by varying its concentration than rice gels. The exponents *n* and q of millet and sorghum were notably higher than that of fonio; however, their viscoelastic moduli at low concentrations (quantified by the m and p coefficients) were much lower than those obtained for fonio. The combination of a high consistency at low concentrations (compared to millet, sorghum, and maize) and a high increase in consistency with increasing concentration (compared to rice) makes fonio an interesting ingredient for the production of GF products of gel-like nature.

### 3.5. Thermal Properties

Thermal properties of the gluten-free flours were evaluated using Differential Scanning Calorimetry (DSC). Gelatinization, retrogradation, and amylose-lipid complex data obtained from DSC are shown in Table 5. Thermograms of flour samples showed 2 wide endothermic transitions in the first scan; one due to starch gelatinization, which appeared at 73–77 °C, and the second peak at 93–98 °C, due to the dissociation of amylose-lipid complex [43]. The reversibility of this second endotherm indicates the presence of an amorphous amylose-lipid complex within these samples [44]. The different flour samples showed significant differences in gelatinization enthalpy (ΔH_gel_). Fonio and rice gels presented the highest values (11.5 and 10.5 J/g, respectively), while maize presented the lowest (5.1 J/g). Higher values of ΔH_gel_ are indicative of a better-packed starch structure requiring more energy to fully gelatinize. Significant differences were also found among gelatinization temperatures. Ji et al. [45] discovered that higher crystallites perfection is linked to greater gelatinization temperatures required to melt them. Fonio presented a high value of onset temperature (To), statistically equal to that of rice, surpassed by millet and sorghum. Peak temperature (Tp), where the endothermic transition reaches a maximum, presented the highest value in millet flour (76.98 °C), while the lowest values were recorded in fonio and maize flours, being 73.5 and 70.07 °C, respectively. The width of the gelatinization temperature range (Te-To) was the highest in maize (17.3 °C), followed by rice (11.3 °C), sorghum (10.6 °C), fonio (9.96 °C), and millet (7.45 °C). Lower values of the gelatinization peak width indicate higher starch crystallites homogeneity and a better organized granular structure, requiring a shorter temperature range to fully hydrate. The formation of amylose–amylose linkages and amylose–lipid complexes within the starch granule and a more stable configuration have been associated with higher gelatinization temperatures [46]. The dissociation enthalpy of amylose–lipid inclusion complex obtained in the first run for fonio was equal to those of millet, sorghum, and rice.

A second scan was performed to assess the retrogradation properties of the flours after storage of the gelatinized samples in their corresponding pans at 4 °C for 7 days (Table 5). Two visible peaks were detected from this scan. The first peak, which appeared at a temperature around 50 °C, was linked to the melting of the recrystallized amylopectin (ΔH_ret_) during the gel staling. All values determined for ΔH_ret_ were lower than their corresponding ΔH_gel_ value, but showed the same trend, with fonio presenting the highest value. The width of this first peak was observed to be higher in fonio flour, while maize presented the lowest value. The second peak registered was related to the reversible amylose–lipid complex dissociation and appeared approximately at the same temperature as it did in the first scan. The enthalpies of the amylose–lipid complex were increased in the second scan with respect to the first scan. Eliasson [44] indicated that the increased values during the second scan are due to better conditions for complex formation after the first heating. This is related to the leaking of amylose out of the granules that occur at temperatures above the gelatinization temperature range.

### 3.6. Texture of Fresh Gel and Its Evolution with Storage Time

**Figure 2:**
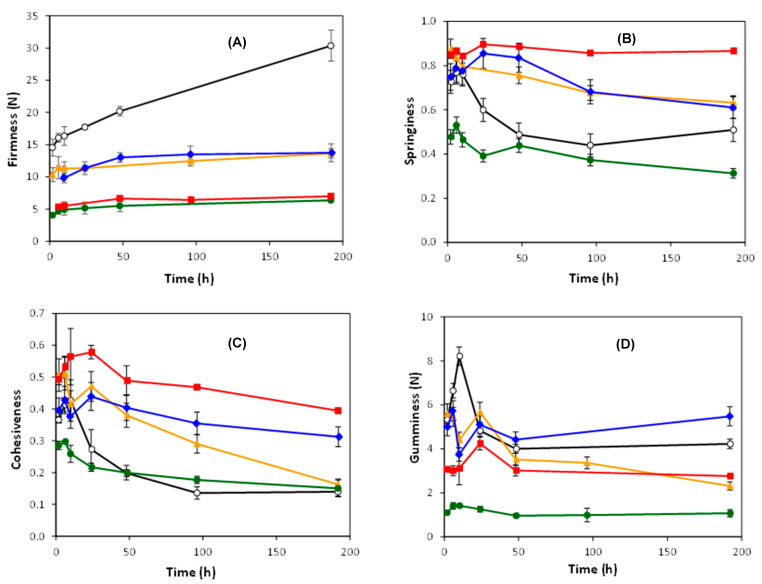
Evolution of firmness (**A**), springiness (**B**) cohesiveness (**C**) and gumminess (**D**) of gels made from fonio (○), millet (▲), sorghum (♦), maize (●) and rice (■) flours at 15% concentration with the storage time at 4 ± 2 °C. The error bars represent the standard deviation.

The texture properties of the gels made from the studied flours and their evolution during a period of 192 h of storage at 4 °C, depicted in Figure 2 and Table 6. Fonio gels presented the highest values of firmness in the studied range, in agreement with the information obtained from gel’s rheology. As could be expected, the firmness of gels increased with storage time regardless of the type of flour. As can be seen in Figure 2A, except for the fonio gel, all gels reached a constant/asymptotic firmness value after 24–48 h of storage. In the case of fonio, however, a constant increase was obtained during all 192 h, reaching a value of 30.37 N, representing an increase of 109% with respect to the initial firmness. This change over storage periods was mainly related to amylopectin recrystallization [47], hence highly dependent on the starch composition and botanical origin of the sample.

Springiness is a mechanical textural attribute related to the rapidity and degree of recovery from a deforming force [48]. The initial springiness of fonio gel was lower than those gels made from the remaining flours, except for maize. Gels’ springiness decreased significantly (*p* < 0.05) with storage time, except for rice gel which showed a very stable springiness value during the whole studied period. Fonio was the only sample to show some springiness recovery after 100 h, with the value corresponding to 192 h being higher than those determined at 48 and 96 h. In general, the cohesiveness of all gels showed a similar trend to that obtained for springiness, with decreasing values during the studied storage time.

The value of gumminess increased after 6 h and decreased at longer times. The highest value was recorded in fonio and millet gels, but after 192 h, the fonio gel had only lost 21% of this maximum value, while millet lost 59%. This parameter is often used to characterize the necessary energy to disintegrate semisolid foods and is adequately correlated to sensory evaluation by a trained panel [49].

## 4. Conclusions

Fonio flour demonstrated good performance when comparing its properties to other gluten-free sources, showing significant differences in techno-functional, pasting, rheological, and gelling properties. Fonio presented a higher ability to absorb water during gelatinization and swelling in excess water, resulting in the highest determined values of WAI and SP, believed to be influenced by its small starch granule size and high specific surface area. This greater ability to interact with water was also verified in the evaluated gel properties. Fonio had a higher pasting viscosity, as well as high shearing and heating stability and resistance, consistent with a better-packed starch structure than the other analyzed GF flours, as shown by the thermal analysis. Rheological properties revealed that fonio flour generated gels with a remarkably strong structure, particularly at high flour concentrations (12% and 15%). Fonio gels also exhibited notably higher elastic modulus and firmness values than the rest of the GF flours. These combined results lead to the conclusion that fonio has the potential to be used in food, especially in preparations such as porridge, where better gelling properties are appreciated. Therefore, it is suggested that fonio is a promising starch source to compete with other commercially important flours, such as rice, in industrial applications.

## Figures and Tables

**Table 1:** Proximal composition of fonio compared to the other gluten-free flours. Except for the moisture content, all presented values are referred to dry basis.

Flour	Moisture (%)	Ash (%)	Fat (%)	Proteins (%)	Carbohydrates (%)
Fonio	12.90 ± 0.01 e	0.45 ± 0.02 a	1.1 ± 0.2 a	6.6 ± 0.3 a	91.9 ± 0.9 c
Millet	10.47 ± 0.01 a	1.73 ± 0.09 e	4.2 ± 0.8 c	12.9 ± 0.5 d	81.2 ± 1.4 a
Sorghum	11.77 ± 0.01 b	1.35 ± 0.07 d	3.3 ± 0.6 b	10.0 ± 0.4 c	85.3 ± 1.1 b
Maize	11.96 ± 0.01 c	1.14 ± 0.06 c	4.4 ± 0.9 c	7.5 ± 0.3 b	87.0 ± 1.3 b
Rice	12.66 ± 0.01 d	0.57 ± 0.03 b	1.3 ± 0.2 a	7.8 ± 0.3 b	90.4 ± 0.6 c

Data are the mean (*n* = 2) ± standard deviation. Values with a letter in common in the same column are not significantly different (*p* > 0.05).

**Table 2:** Functional characteristics and pasting properties of fonio flour compared to other gluten-free flours.

	Fonio	Millet	Sorghum	Maize	Rice
Functional properties					
FC (mL)	nd	3.33 ± 0.34 b	4.99 ± 0.35 c	1.57 ± 0.17 a	1.74 ± 0.01 a
FS (%)	nd	52 ± 9 a	82 ± 2 c	76 ± 8 b	50 ± 1 a
WAC (g/g)	1.12 ± 0.02 c	0.97 ± 0.01 a	1.09 ± 0.01 b	1.34 ± 0.02 d	0.98 ± 0.01 a
WHC (g/g)	1.8 ± 0.2 bc	1.8 ± 0.1 abc	1.85 ± 0.07 c	2.4 ± 0.1 d	1.65 ± 0.09 a
WAI (g/g)	7.7 ± 0.2 d	6.20 ± 0.07 b	6.9 ± 0.3 c	6.35 ± 0.06 b	5.77 ± 0.03 a
WSI (g/100 g)	0.7 ± 0.1 a	2.2 ± 0.1 cb	2.2 ± 0.1 c	4.7 ± 0.1 d	1.74 ± 0.03 bc
SP (g/g)	7.7 ± 0.2 d	6.16 ± 0.07 b	6.9 ± 0.3 c	6.26 ± 0.06 b	5.74 ± 0.03 a
Pasting properties					
PT (°C)	82.2 ± 0.1 b	85.3 ± 0.1 d	91.6 ± 0.2 e	80.29 ± 0.02 a	82.72 ± 0.02 c
Pt (s)	562 ± 3 a	620 ± 1 c	682 ± 6 e	572 ± 5 b	633 ± 1 d
PV (Pa·s)	4.68 ± 0.03 d	2.11 ± 0.03 b	2.56 ± 0.03 c	1.580 ± 0.004 a	5.22 ± 0.09 e
FV (Pa·s)	5.15 ± 0.08 c	4.46 ± 0.02 b	5.4 ± 0.4 c	2.92 ± 0.02 a	5.22 ± 0.09 c
TV (Pa·s)	2.39 ± 0.02 d	1.32 ± 0.01 a	2.00 ± 0.02 b	1.270 ± 0.001 a	2.19 ± 0.04 c
BV (Pa·s)	2.29 ± 0.01 d	0.79 ± 0.04 c	0.56 ± 0.05 b	0.310 ± 0.005 a	3.03 ± 0.05 e
SV (Pa·s)	2.76 ± 0.07 b	3.14 ± 0.02 bc	3.4 ± 0.5 c	1.65 ± 0.02 a	3.03 ± 0.05 bc

FC: foaming capacity, FS: foaming stability, WHC: Water holding capacity (g H_2_O/g flour dry matter, dm), WAI: Water absorption index (g sediment/g flour dm), WSI: Water solubility index (g soluble solids/100 g flour dm), WAC: Water absorption capacity (g H_2_O/g flour dm), SP: swelling power (g/g insoluble flour matter). PT: Pasting Temperature, Pt: peak time, PV: peak viscosity, FV: final viscosity, TV: Trough Viscosity, BV: Breakdown viscosity, SV: Setback viscosity. Data are mean ± standard deviation. Values with a letter in common in the same row are not significantly different (*p* > 0.05).

**Table 3:** Rheological properties of fonio, millet, sorghum, maize, and rice flour gels at different concentrations.

Flour	Concentration (%)	G_1_′ (Pa)	a	G_1_″ (Pa)	B	tan (δ)_1_	C	Cross over (Pa)	τ_max_ (Pa)
Fonio	6	45 ± 4 aA	0.121 ± 0.006 eC	10 ± 1 aB	0.370 ± 0.007 dC	0.230 ± 0.007 cD	0.249 ± 0.001 abA	14.5 ± 0.4 aC	8.3 ± 0.1 aC
	8	385 ± 3 bD	−0.134 ± 0.006 aA	30 ± 1 bC	0.267 ± 0.001 cA	0.078 ±0.001 bA	0.401 ± 0.005 bD	574 ± 26 bD	548 ± 18 bD
	10	1242 ± 10 cC	−0.072 ± 0.002 bA	70 ± 1 cC	0.184 ± 0.001 aA	0.056 ± 0.004 aA	0.256 ± 0.003 abD	1347 ± 28 cD	1142 ± 4 cD
	12	1761 ± 66 dE	−0.003 ± 0.003 cA	95 ± 3 dD	0.201 ± 0.002 bC	0.054 ± 0.003 aA	0.204 ± 0.001 aB	1682 ± 102 dD	1387 ± 37 dD
	15	3106 ± 182 eE	0.0150 ± 0.0003 dA	169 ± 11 eBC	0.209 ± 0.005 bC	0.055 ± 0.004 aA	0.193 ± 0.005 aC	2535 ± 4 eD	2151 ± 76 eE
Millet	6	66 ± 5 aBC	0.0320 ± 0.0001 aA	6 ± 1 aA	0.356 ± 0.004 dB	0.099 ± 0.002 cA	0.324 ± 0.003 cC	11.0 ± 0.5 aA	3.9 ± 0.3 aB
	8	167 ± 11 aA	0.064 ± 0.001 eB	17 ± 1 aA	0.290 ± 0.01 cB	0.105 ± 0.003 dB	0.230 ± 0.013 bBC	34 ± 2 aA	12 ± 3 abA
	10	380 ± 36 bB	0.059 ± 0.003 dB	38 ± 3 bA	0.219 ± 0.014 bB	0.101 ± 0.002 cB	0.160 ± 0.010 aB	96 ± 14 bA	29 ± 2 bA
	12	833 ± 31 cC	0.047± 0.003 cB	71 ± 2 cB	0.181 ± 0.009 aB	0.086 ± 0.001 bB	0.133 ± 0.006 aA	204 ± 27 cA	95 ± 4 cA
	15	2213 ± 107 dC	0.0410 ± 0.0003 bB	176 ± 11 dC	0.189 ± 0.005 aB	0.079 ± 0.001 aB	0.148 ± 0.005 aB	526 ± 49 dBC	245 ± 11 dC
Sorghum	6	59 ± 2 aBC	0.043 ± 0.006 aA	6 ± 1 aA	0.363 ± 0.001 dBC	0.101 ± 0.003 aA	0.321 ± 0.006 bB	9.0 ± 0.3 aA	2.0 ± 0.1 aA
	8	202 ± 5 bB	0.079 ± 0.003 dB	27 ± 1 bB	0.240 ± 0.007 cC	0.132 ± 0.001 dC	0.160 ± 0.010 aA	36.6 ± 0.5 bAB	11 ± 2 bA
	10	458 ± 7 cB	0.068 ± 0.001 cBC	56 ± 1 cB	0.185 ± 0.003 bA	0.121 ± 0.003 cC	0.116 ± 0.004 aA	83 ± 1 cA	28.1 ± 0.2 cA
	12	1059 ± 19 dD	0.059 ± 0.002 bC	112 ± 4 dD	0.168 ± 0.001 aA	0.106 ± 0.001 bC	0.109 ± 0.001 aA	194 ± 12 dA	68 ± 6 dA
	15	2671 ± 4 eD	0.056 ± 0.001 bD	280 ± 5 eD	0.179 ± 0.004 bA	0.105 ± 0.002 bC	0.123 ± 0.005 aA	438 ± 6 eA	120.3 ± 0.3 eA
Maize	6	54 ± 1 aB	0.086 ± 0.003 cB	10 ± 1 aB	0.311± 0.001 eA	0.187 ± 0.004 eC	0.225 ± 0.003 aA	13.8 ± 0.7 aB	3.6 ± 0.3 aB
	8	226 ± 6 bC	0.07 ± 0.02 bcB	36 ± 2 bD	0.242 ± 0.001 dC	0.158 ± 0.003 dE	0.170 ± 0.020 aAB	59 ± 3 bBC	34.6 ± 0.4 aB
	10	407 ± 69 cB	0.062 ± 0.007 abBC	57 ± 11 cB	0.220 ± 0.004 cB	0.139 ± 0.003 cD	0.158 ± 0.005 aB	153 ± 2 cB	85 ± 6 bB
	12	673 ± 9 dB	0.051 ± 0.001 abB	812 ± 2 dE	0.209 ± 0.004 bC	0.121 ± 0.002 bD	0.158 ± 0.004 aA	364 ± 1 dC	292 ± 2 cC
	15	1492 ± 6 eB	0.044 ± 0.001 aC	155 ± 1 eB	0.193 ± 0.006 aB	0.104 ± 0.001 aC	0.149 ± 0.006 aB	759 ± 8 eC	454 ± 3 dD
Rice	6	93 ± 1 aD	0.087 ± 0.001 cB	15 ± 1 aC	0.314± 0.001 bA	0.156 ± 0.003 bB	0.227 ± 0.001 bA	36 ± 2 aC	22.7 ± 0.7 aD
	8	174 ± 3 bA	0.0530 ± 0.0001 aB	25 ± 1 bB	0.305 ± 0.001 aD	0.143 ± 0.001 aD	0.252 ± 0.001 dC	118 ± 1 bC	60.4 ± 0.7 bC
	10	196 ± 2 cA	0.072 ± 0.002 bD	32 ± 1 cA	0.321 ± 0.001 cC	0.164 ± 0.003 cE	0.249 ± 0.003 cC	206 ± 2 cC	97.3 ± 0.2 cC
	12	225 ± 4 dA	0.088 ± 0.003 cD	40 ± 1 dA	0.334 ± 0.001 dD	0.180 ± 0.001 dE	0.25 ± 0.02 bC	302 ± 4 dB	154 ± 1 dB
	15	338 ± 5 eA	0.121 ± 0.001 dE	61 ± 1 eA	0.341 ± 0.001 eD	0.181 ± 0.001 dD	0.220 ± 0.002 aD	460 ± 9 eB	217 ± 4 eB

The power-law model was fitted to experimental results from frequency sweeps: G′(ω)= G_1_′·ω^a^; G″(ω)= G_1_″·ω^b^; tan δ (ω)= tan δ_1_·ω^c^. G_1_′, G_1_″ and (tan δ)_1_: represent the elastic and viscous moduli and the loss tangent at 1% strain. The a, b, and c exponents quantify the dependence degree of dynamic moduli and the loss tangent with the oscillation frequency. τ_max_: maximum stress that samples can tolerate in the LVR. Data are the mean ± standard deviation. Values with a letter in common in the same column for each flour are not significantly different (*p* > 0.05). Lowercase letters compare the effect of concentration in the same flour, and capital letters compare different flours at the same concentration.

**Table 4:** Parameters obtained from fitting to the power-law model the experimental G_1_′ and G_1_″ data in function of the flour concentration in the gels (G_1_′ = m·C^n^; G_1_″ = p·C^q^).

Parameter	Fonio	Millet	Sorghum	Maize	Rice
m (Pa)	2 ± 1 d	0.015 ± 0.004 a	0.030 ± 0.005 b	0.20 ± 0.08 c	11 ± 5 e
*n*	2.8 ± 0.3 b	4.4 ± 0.1 e	4.21 ± 0.06 d	3.3 ± 0.1 c	1.26 ± 0.17 a
R^2^	0.979	0.9992	0.9997	0.9971	0.9561
p (Pa)	0.18 ± 0.09 b	0.005 ± 0.002 a	0.006 ± 0.001 a	0.15 ± 0.06 b	1.0 ± 0.2 c
q	2.5 ± 0.2 b	3.9 ± 0.1 c	4.00 ± 0.07 c	2.6 ± 0.2 b	1.53 ± 0.09 a
R^2^	0.989	0.9985	0.9995	0.9934	0.9914

The coefficients m and p represent the values of the G_1_′ and G_1_″ moduli at a concentration of 1%; *n* and q exponents inform about the dependence degree of both moduli on flour concentration. Values with a letter in common in the same row are not significantly different (*p* > 0.05).

**Table 5:** Thermal properties of fonio, millet, sorghum, maize, and rice flours.

	First Scan					Second Scan						
Samples	ΔH_gel_ (J/g)	To_gel_ (°C)	Tp_gel_ (°C)	Te_gel_ (°C)	ΔH_am-lip_ (J/g)	Tp_am-lip_ (°C)	ΔH_ret_ (J/g)	To_ret_ (°C)	Tp_ret_ (°C)	Te_ret_ (°C)	ΔH_am-lip_ (J/g)	Tp_am-lip_ (°C)
Fonio	11.5 ± 0.3 e	68.92 ± 0.03 b	73.5 ± 0.1 b	78.88 ± 0.08 b	0.91 ± 0.01 b	97.0 ± 0.2 bc	7.9 ± 0.3 b	32 ± 6 a	50.5 ± 0.3 a	68 ± 4 b	1.3 ± 0.2 a	97.8 ± 0.1 c
Millet	7.0 ± 0.1 c	73.45 ± 0.01 d	76.98 ± 0.02 d	80.9 ± 0.1 d	0.94 ± 0.05 b	93.3 ± 0.1 a	4.5 ± 0.4 a	38 ± 1 a	52.1 ± 0.1 b	63.5 ± 0.3 ab	3.1 ± 0.2 bc	92.6 ± 0.3 a
Sorghum	6.5 ± 0.4 b	69.8 ± 0.1 c	74.7 ± 0.1 c	80.4 ± 0.2 cd	0.99 ± 0.06 b	98.1 ± 0.2 c	5.1 ± 0.6 a	36 ± 2 a	51 ± 1 a	64.2 ± 0.3 ab	3.6 ± 0.7 c	95 ± 1 b
Maize	5.1 ± 0.5 a	61.1 ± 0.1 a	70.07 ± 0.01 a	78.4 ± 0.3 a	0.16 ± 0.03 a	97 ± 1 bc	4.3 ± 0.1 a	38 ± 1 a	50.7 ± 0.8 ab	62.6 ± 0.2 a	2.0 ± 0.2 a	91.6 ± 0.6 a
Rice	10.5 ± 0.2 d	68.9 ± 0.2 b	74.68 ± 0.06 c	80.2 ± 0.2 c	0.98 ± 0.05 b	96.5 ± 0.2 b	5.2 ± 0.6 a	39 ± 1 a	50.4 ± 0.1 a	62.8 ± 0.5 ab	2.3 ± 0.4 ab	98.0 ± 0.1 c

ΔH_gel_, ΔH_am-lip,_ and ΔH_ret_: Enthalpy associated to starch gelatinization, dissociation of amylose lipid complex and melting of the recrystallized amylopectin; To_gel_, To_ret_: onset temperature of gelatinization and retrogradation peaks; Tp_gel_, Tp_ret_, Tp_am-lip_: Peak Temperature of gelatinization, retrogradation, and amylose-lipid complex dissociation peaks; Te_gel_, Te_ret_: endset temperature of gelatinization and retrogradation peaks; First scan: Scan carried out on native (un-gelatinized) sample. Second scan: scan carried out on gelatinized samples after 7 days of storage at 4 °C. Means values with different letters for the same parameter imply significant differences between means at *p* < 0.05.

**Table 6:** Textural parameters of fresh gels (15% flour concentration) and their evolution with storage at 4 °C for 192 h (8 days).

Parameter	Fonio	Millet	Sorghum	Maize	Rice
Firmness (N)					
P_0_	15 ± 1 d	11 ± 1 c	12.7 ± 0.4 cd	3.9 ± 0.3 a	6.2 ± 0.1 b
ΔP (%)	109 ± 17 c	27 ± 6 a	9 ± 11 a	65.0 ± 0.6 b	12 ± 4 a
Springiness					
P_0_	0.73 ± 0.01 b	0.88 ± 0.01 c	0.75 ± 0.03 b	0.48 ± 0.05 a	0.85 ± 0.01 c
ΔP (%)	−30 ± 10 a	−28 ± 13 a	−7 ± 16 ab	−34 ± 11 a	−2 ± 1 b
Cohesiveness					
P_0_	0.37 ±0.01 b	0.51 ±0.04 c	0.40 ± 0.03 b	0.29 ± 0.01 a	0.49 ± 0.02 c
ΔP (%)	−62 ± 4 a	−68 ± 4 a	−21 ± 21 b	−47 ± 9 ab	−20.0 ± 0.7 b
Gumminess (N)					
P_0_	5.3 ± 0.6 c	5.60 ± 0.06 c	5.0 ± 0.5 c	1.10 ± 0.03 a	3.08 ± 0.08 b
ΔP (%)	−21 ± 2 ab	−59 ± 8 a	−13 ± 32 b	−13 ± 15 b	−10 ± 2 b
Resilience					
P_0_	0.17 ± 0.01 b	0.36 ± 0.05 d	0.21 ± 0.02 bc	0.09 ± 0.01 a	0.23 ± 0.02 c
ΔP (%)	−57 ± 11 a	−75 ± 4 a	−13 ± 33 b	−41 ± 17 ab	−40 ± 5 ab

ΔP = 100·(P_8_ − P_o_)/P_o_, P = textural parameter, P_8_ = textural value after 8 d of storage, P_0_ = initial textural value. Values with a letter in common in the same row are not significantly different (*p* > 0.05).

## Data Availability

The data presented in this study are available on request from the corresponding author.

## Supplementary Materials

The following are available online at https://www.mdpi.com/article/10.3390/foods11020183/s1, Figure S1: Evolution of the elastic modulus (A), viscous modulus (B), and of the loss tangent (C) of gels.
