# Supplementary material for: Techno-Functional and Gelling Properties of Acha (Fonio) (Digitaria exilis stapf) Flour: A Study of Its Potential as a New Gluten-Free Starch Source in Industrial Applications
Source: Foods. 2022 Jan 11;11(2):183. doi: 10.3390/foods11020183 (PMC8774706; doi:10.3390/foods11020183)
Supplement: Supplementary file 1 [file foods-11-00183-s001.zip › foods-1501597-supplementary.pdf]

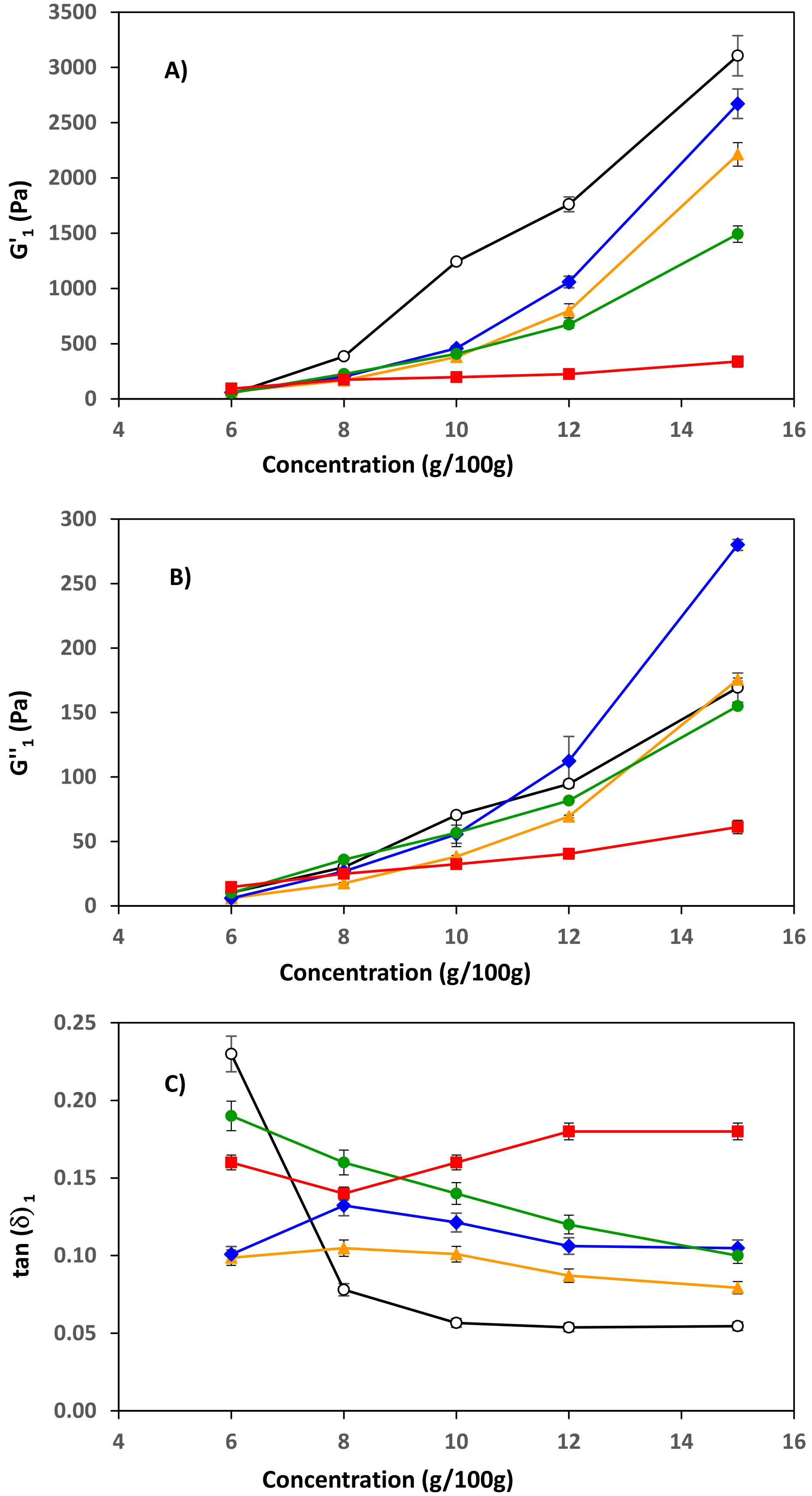

Figure S1. Evolution of the elastic modulus (A), viscous modulus (B) and of the loss tangent (C) of gels made from fonio (○), millet (▲), sorghum (◆), maize (●) and rice (■) flours versus gel concentration. The error bars represent the standard deviation.
